# A Preference for Contralateral Stimuli in Human Object- and Face-Selective Cortex

**DOI:** 10.1371/journal.pone.0000574

**Published:** 2007-06-27

**Authors:** Christopher C. Hemond, Nancy G. Kanwisher, Hans P. Op de Beeck

**Affiliations:** 1 McGovern Institute for Brain Research and Department of Brain & Cognitive Sciences, Massachusetts Institute of Technology, Cambridge, Massachusetts, United States of America; 2 Martinos Center for Biomedical Imaging, Charlestown, Massachusetts, United States of America; 3 Laboratory of Experimental Psychology, University of Leuven (K.U.Leuven), Leuven, Belgium; Université di Parma, Italy

## Abstract

Visual input from the left and right visual fields is processed predominantly in the contralateral hemisphere. Here we investigated whether this preference for contralateral over ipsilateral stimuli is also found in high-level visual areas that are important for the recognition of objects and faces. Human subjects were scanned with functional magnetic resonance imaging (fMRI) while they viewed and attended faces, objects, scenes, and scrambled images in the left or right visual field. With our stimulation protocol, primary visual cortex responded only to contralateral stimuli. The contralateral preference was smaller in object- and face-selective regions, and it was smallest in the fusiform gyrus. Nevertheless, each region showed a significant preference for contralateral stimuli. These results indicate that sensitivity to stimulus position is present even in high-level ventral visual cortex.

## Introduction

In the primate visual system visual input is processed predominantly in the contralateral hemisphere. This characteristic of visual processing is very strong in the primary visual cortex (V1), where input from the left visual field is processed almost exclusively in the right hemisphere and vice versa. This contralateral preference decreases at higher levels of the object vision pathway in ventral visual cortex, as do other aspects of retinotopic organization [1]. Parallel to this decrease in contralateral preference, there is an increase in the tolerance to a range of image manipulations, like changes in position, size and orientation [2], [3].

Traditionally, object- and face-selective regions in both the lateral occipital gyrus (e.g., “lateral occipital” or LO; “occipital face area” or OFA) and the fusiform gyrus (e.g., “posterior fusiform” or PF: “fusiform face area” or FFA) have been considered “non-retinotopic” regions. Several studies used fMRI adaptation to assess the sensitivity of these regions to manipulations of object identity, orientation, size, and position [4]-[8]. Object- and face-selective regions showed less sensitivity to these manipulations than retinotopic areas, and the regions in the fusiform gyrus tended to show less sensitivity than the object- and face-selective regions in lateral occipital gyrus. Nevertheless, even regions in fusiform gyrus still showed some sensitivity to various image manipulations like stimulus position [4].

These studies measured sensitivity to location indirectly through the effect of image manipulations on the amount of adaptation. Contralateral preference is one aspect of position sensitivity that can be studied directly because neuronal populations with different position preferences are generally anatomically segregated at a resolution that is easily measurable with fMRI. Recently, a clear contralateral preference has been reported in lateral occipital cortex [9], but no contralateral preference has been reported in the object- and face-selective regions in the middle fusiform gyrus. Here we show that although these object- and face-selective regions in the fusiform gyrus show a weaker contralateral preference than lateral occipital regions, the effect is significant in each of these regions.

## Materials and Methods

### Subjects

Nine subjects (all right-handed; three males) participated in this experiment. Subjects were college or graduate students in the Boston area, and all of them were healthy, paid volunteers. Informed consent was obtained and all procedures were approved by the Institutional Review Boards of Massachusetts Institute of Technology and Massachusetts General Hospital.

### Stimulus presentation

**Figure 1:**
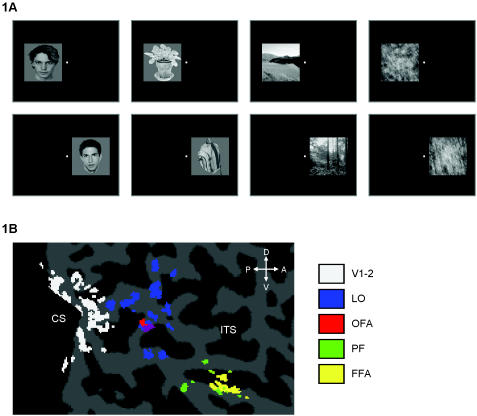
Illustration of the experimental conditions and the anatomical position of the regions of interest (ROIs). (A), Illustration of the position of the four stimulus categories (faces, objects, scenes, and scrambled images) left and right of the fixation spot. (B) Illustration of the 5 ROIs for one subject onto a flattened brain. Indicated sulci: CS: calcarine sulcus; ITS: inferior temporal sulcus. Indicated anatomical directions: D: dorsal; V: ventral; P: posterior; A: anterior.

Stimuli were presented in 15-second blocks of fixation, human faces, objects, outdoor scenes, and Fourier-scrambled images. Stimulus duration was 300 ms with 450 ms inter-stimulus interval (20 stimuli per 15-second block). There were four 15-second blocks for each stimulus category in each fMRI time series and 20 different stimuli per condition (each presented once in each block). The fixation dot was 0.2 × 0.2 degrees of visual angle, and it was always present in each condition. Examples of stimuli and their arrangement on the screen are shown in Figure 1A. The size of the rectangle containing each stimulus was 8 × 8 visual degrees; visual information for the scenes and the Fourier-scrambled images covered this area entirely, but that was not true for most faces and objects (see Fig. 1). The background within each stimulus rectangle was slightly brighter than the rest of the screen, so the faces and the objects were shown inside a visible square. In each block, stimulus position was either in the left visual field or the right visual field. The closest border of the stimulus area was 1 visual degree from the center of the fixation spot, with a small jitter in the vertical stimulus position of maximum 2 degrees from the horizontal midline.

Subjects were instructed to perform a demanding color change detection task during stimulus presentation while holding fixation on the central fixation spot. This task required subjects to press a key each time an object had a different color than the previous object (3 changes in each block of 20 stimuli). Low-saturated color was added to the grey-scale images by increasing the value of one color channel and decreasing the value of the other channels by a factor c. This parameter was the same for all conditions in a run, but it was adapted between runs to keep the task demanding for the subjects. This task assures that subjects were attending to the non-foveal stimuli. The need to fixate the fixation spot was mentioned at the start of each time series. Fixation quality was not verified on-line with eye-tracking devices, but the absence of significant activation in ipsilateral V1 indicates that fixation performance was very good (see Results).

### Scanning parameters

Subjects were scanned in two sessions. Scanning was carried out at the Martinos Center for Biomedical Imaging at Massachusetts General Hospital in a 3T Siemens Trio magnet with an 8-channel phased-array head coil (Siemens). Functional images were acquired with an EPI sequence including an integrated Parallel Acquisition Technique (105 time points per time series; TR  =  3 s; TE  =  37 ms; 128 × 128 matrix; 1.4 × 1.4 mm in-plane voxel size; 20 slices approximately perpendicular to the calcarine sulcus covering the entire occipital and occipitotemporal cortex with slice thickness 2 mm and inter-slice gap 0.4 mm). In each session, we also acquired a T1-weighted anatomical image. We made sure that the head position and slice prescription were very similar in the two scan sessions.

In total we acquired 8 time series with lateralized stimulus presentation. These time series were interleaved with other time series with other stimulus conditions of which the data have been published elsewhere [10], and for which the lateralized stimuli served as localizer data. The data from the other time series are irrelevant for the purposes of the present paper.

### Analysis of imaging data

Data were analyzed with FS-FAST, Freesurfer (http://surfer.nmr.mgh.harvard.edu/) [11], [12], froi (http://froi.sourceforge.net), as well as custom Matlab code. Pre-processing involved motion correction, smoothing with a Gaussian kernel of 3 mm FWHM, and normalization. The pre-processing did not involve any spatial normalization of subjects in a common reference space (e.g., Talairach transformations). The functional data of the two sessions of each subject were co-aligned directly (without an intermediate step through anatomical data) by aligning all data to the first functional image of the first scan session. This functional reference image was co-registered with that subject's anatomical image.

The predictor for each stimulus condition (zero or one at each timepoint) was convolved with a gamma function, and the general linear model was used to compute the response of each voxel in each condition. The response for each condition in each voxel is expressed in units of percent signal change (PSC), which is the response in each condition minus the response in the fixation condition, normalized by the mean signal value at each voxel. Significance maps of the brain were computed by performing *t*-tests for pair-wise comparisons of conditions, and thresholded at *p*  =  0.0001 (uncorrected for multiple comparisons). We used this same statistical threshold throughout all analyses to define regions of interest.

We investigated 5 regions of interest (ROIs) in each hemisphere of each subject (illustrated for one subject in Figure 1B):

1. V1-2 (early visual cortex; average of 103 voxels per subject): All visually responsive voxels (significantly higher response in blocks with visual stimuli compared to fixation blocks) around the posterior tip of the calcarine sulcus. This ROI includes the foveal and parafoveal representation of primary visual cortex (V1) and possibly also part of secondary visual area V2. This ROI was defined in the right hemisphere for all subjects, and in the left hemisphere for 8 out of 9 subjects (the occipital pole of the left hemisphere was not covered by the slice prescription in one subject).
2. LO (lateral occipital; 559 voxels): Voxels that were significantly activated in the contrast [objects – scrambled], and located around the lateral occipital gyrus. This ROI was defined in each hemisphere for each subject.
3. OFA (occipital face area; 64 voxels): Voxels that were significantly activated in the contrast [faces-objects], and located around the lateral occipital gyrus. This ROI was defined in the right hemisphere for all subjects, and in the left hemisphere for 5 out of 9 subjects (the other 4 subjects did not show significant face-selective responses in the left lateral occipital gyrus).
4. PF (posterior fusiform; 87 voxels): Voxels that were significantly activated in the contrast [objects – scrambled], and located around the fusiform gyrus. This ROI was defined in each hemisphere for each subject.
5. FFA (fusiform face area; 62 voxels): Voxels that were significantly activated in the contrast [faces-objects], and located around the fusiform gyrus. This ROI was defined in each hemisphere for each subject. A more restricted FFA was also defined as the one voxel having the average coordinates of all FFA voxels, or, in case the activated voxels in the fusiform gyrus formed more than one homogenous patch, the average coordinates of the largest patch.

It is important to note that each ROI was defined across both ipsilateral and contralateral stimulus presentation. Thus, the ROI definition is orthogonal to the contralaterality question. Of course, the ROI definition is not orthogonal to the overall level of visual activation (for V1) or the selectivity for objects (for LO and PF) or faces (for OFA and FFA). Thus, the amount of object or face selectivity in these regions might be slightly over-estimated.

The reported contralateral preferences are averaged across the left and right hemispheres, taking the values of only one hemisphere in case the ROI was not defined in one of the hemispheres of a subject. Most ROIs showed a significant contralateral bias in each hemisphere, so the average effects that we report here are found in each hemisphere. The only exception was PF, as mentioned in the Results section.

### Preference index

The contralateral preference in each ROI was quantified for each subject and each stimulus condition as (response to contralateral stimuli – response to ipsilateral stimuli) / (response to contralateral stimuli). Thus a higher preference index signifies a stronger contralateral preference.

## Results

**Figure 2:**
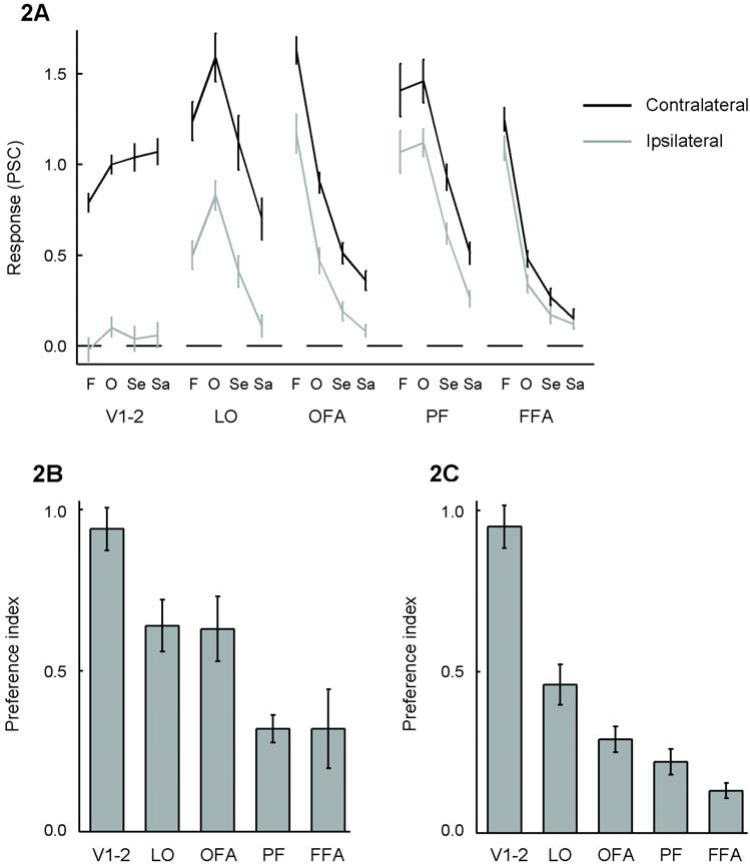
Responses to contralateral and ipsilateral stimuli in the regions of interest. (A) The response (percent signal change relative to the fixation condition) in each ROI is shown for each stimulus condition (F: faces, O: objects, Se: scenes, Sa: scrambled images). (B) Preference index in each ROI averaged across all stimulus conditions. (C) Preference index in each ROI for the stimulus condition that elicited the strongest responses. Error bars represent the standard error of the mean across subjects.

Figure 2A shows the contralateral and the ipsilateral responses for each stimulus category in each of the ROIs. These data are summarized by the preference index averaged across all stimulus categories (Figure 2B) and for the preferred stimulus category (Figure 2C).

### Primary visual cortex (V1-2)

As expected from previous studies, there was a strong contralateral preference in V1-2. The preference index was significantly different from zero across subjects (*P* < 0.00001, *t*-test) when averaged across all stimulus categories and also for the stimulus category that elicited the strongest response (scrambled images). The contralateral preference was absolute, in the sense that there was no detectable response to ipsilateral stimuli with our stimulation protocol: There was no significant response to ipsilateral stimuli (relative to fixation) averaged across all stimulus categories (*P*  =  0.50, *t*-test) and for scrambled images (*P*  =  0.39, *t*-test). Likewise, the preference index was not significantly different from 1 averaged across all stimulus categories (*P*  =  0.39) and for scrambled images (*P*  =  0.29).

These results in V1-2 are an important control for eye movements since we did not monitor eye movements. Any eye movements towards the stimuli would cause foveal stimulation and hence decrease differences between contralateral and ipsilateral conditions in how strongly they stimulate the two hemispheres. This would lead to an underestimation of the contralateral preference. Given that we found no detectable response to ipsilateral stimuli in V1-2 with our stimulation protocol, we cannot be underestimating contralateral preference substantially.

### Lateral occipital gyrus (LO and OFA)

LO and OFA also showed a strong contralateral preference (*P* < 0.001, *t*-test) averaged across all stimulus categories and for the preferred stimulus category (LO: objects; OFA: faces). The response to ipsilateral stimuli was about half of the response to contralateral stimuli. This contralateral preference was significantly less than the absolute contralateral preference in V1-V2 (LO: *P*  =  0.002, OFA: *P*  =  0.041, paired *t*-tests across subjects). LO and OFA did not differ in their contralateral preference across all stimulus categories (*P*  =  0.86). The contralateral preference for the preferred stimulus category was different however: The contralateral preference for objects in LO was stronger than the contralateral preference for faces in OFA (*P*  =  0.015, paired *t*-test).

### Fusiform gyrus (PF and FFA)

PF and FFA also showed a contralateral preference averaged across all stimulus categories (PF: *P*  =  0.0008; FFA: *P*  =  0.032, *t*-test) and for the preferred stimulus category (PF for objects: *P*  =  0.0005; FFA for faces: *P*  =  0.0006). Nevertheless, the response to ipsilateral stimuli was more than two thirds of the response to contralateral stimuli. The contralateral preference in PF was significantly less than the contralateral preference in LO (*P*  =  0.0013, paired *t*-test), and the contralateral preference in FFA was significantly less than the contralateral preference in OFA (*P*  =  0.0079). PF and FFA did not differ in their contralateral preference across all stimulus categories (*P*  =  0.94). The contralateral preference for preferred stimuli (i.e., objects) in PF was slightly stronger than the contralateral preference for preferred stimuli (i.e., faces) in FFA (*P*  =  0.086, paired *t*-test).

PF and FFA contained more voxels in the right hemisphere (PF: 122; FFA: 89) than in the left hemisphere (PF: 51; FFA: 35). FFA showed a significant contralateral preference for faces in each hemisphere (FFA right: *P*  =  0.032; FFA left: *P*  =  0.015), while the contralateral preference in PF for objects was only significant in the right hemisphere (PF right: *P*  =  0.00005; PF left: *P*  =  0.36).

The contralateral preference in FFA was also found with a more restricted ROI including only the FFA voxel with average coordinates, as such excluding the voxels at the border of the ROI where the signal might partially reflect the responses of nearby non-FFA areas (“partial voluming”). In this restricted FFA, the preference index for faces was 0.15 (significantly different from zero: *P*  =  0.006), which is not smaller than the preference index of 0.14 in the larger FFA ROI.

## Discussion

Our data reveal a significant contralateral preference at multiple levels of the human ventral visual pathway. This contralateral preference is weaker in the fusiform gyrus than in the lateral occipital gyrus, confirming findings in the literature indicating that processing in the fusiform gyrus is more invariant to image manipulations like changes in retinotopic position [4]. Nevertheless, even regions in the fusiform gyrus have a preference for contralateral stimuli over ipsilateral stimuli. This was found across all stimulus categories as well as for the preferred category (objects in object-selective cortex and faces in face-selective cortex).

It is important to note that in our design the focus of attention always coincides with the location of the stimuli. A previous study of the contralateral bias in lateral occipital gyrus dissociated the two factors, and the results suggested that both the side of sensory stimulation and the side of attention influence activity in LO [9]. Thus, in our study, the observed contralateral bias probably reflects the combined influence of sensory stimulation and spatial attention. Furthermore, the relative contribution of these two factors might vary across stages of object and face processing.

The strong contralateral bias in lateral occipital cortex is not surprising given the anatomical location and size of this region as well as previous human imaging results. This region is very large (as illustrated in Fig. 1B), and it might be composed of functionally distinct sub-regions, some of which probably overlap with retinotopically organized areas. We defined this area based on a preference for objects over scrambled images, and previous work has shown a preference for intact over scrambled images in V4 [1]. Furthermore, several retinotopic areas have been proposed beyond V4 that might overlap partially with LO and OFA [13], [14]. A previous study that also reported a similar contralateral preference in lateral occipital gyrus claimed that this area did not show any retinotopic organization [9]. It is unclear why some studies report more retinotopically organized regions that others, and many aspects of the stimulation protocols have to be considered (e.g., continuous phase-encoding characterization of the map versus a block design with only a small number of conditions; the extend of the peripheral visual field that is covered; the variation in the stimuli in terms of meaning, color, and motion, etc.). It is possible that all mid-level visual areas show a retinotopic organization, at least weakly, including the contralateral preference reported here.

The contralateral preference in human high-level visual cortex in the fusiform gyrus is a novel finding that has not been demonstrated in previous human imaging studies. Nevertheless, this contralateral preference is consistent with the results from extracellular recordings in the highest-level unimodal region in monkey ventral visual cortex, area TE [15]. The responses of TE neurons provide surprisingly detailed information about the position of stimuli, and their receptive fields for their preferred stimulus have an average diameter of 10 visual degrees (taking as border the position where responses have fallen off 50% compared to the preferred position). Furthermore, there is a clear preference for the contralateral visual field in monkey high-level visual cortex. Calculated with the same method as in the present study, the preference index for TE neurons is approximately 0.33. These data in monkeys were obtained with relatively small stimuli (3.3 visual degrees), and tests with larger stimuli indicated that stimulus size has a substantial effect on estimated receptive field size [16]. Stimuli of 8 degrees as we used here would give larger receptive field estimates and a smaller preference index, estimated to be close to 0.2. This number derived from data in monkeys is within the range of preference indices that we observed in human PF and FFA. If we assume that a similar contralateral preference is a good index for other properties of neuronal receptive fields, then these data suggest that neurons in high levels of the human visual system provide as much information about stimulus position as those found in monkey area TE. This occurrence of position sensitivity in high-level visual cortex seems to go against the hypothesis that the primary function of information processing in the ventral visual stream is the construction of object representations that are invariant to simple image transformations. However, it has been argued recently that such sensitivity to spatial information might be a useful property of object representations, enabling the representation of complex multi-part objects as well as the recognition of objects in cluttered scenes [17], [18].

However, the sensitivity to spatial information might not be the same for all stimulus categories. In the present study, there was a clear effect (*P*  =  0.01) that the contralateral preference for objects across all object-selective regions was stronger than the contralateral preference for faces across all face-selective regions. At the same time, object- and face-selective regions did not differ (*P*  =  0.72) in their contralateral preference across all stimulus categories (compare Fig. 2B and Fig. 2C). These two findings suggest that the object- and face-selective regions receive similar retinotopic inputs and comprise comparable levels in the general visual processing hierarchy, but at the same time the representation of faces in face-selective cortex is more invariant to the stimulated hemifield than the representation of objects in object-selective cortex.

While we focus on a contralateral preference in object- and face-selective regions in this report, it is important to emphasize that unilateral non-foveal stimuli are sub-optimal to activate these regions. Indeed, it has been shown before that face-selective regions are biased to process foveal stimuli during free-viewing [19]. As a consequence, the contralateral bias in face-selective cortex might be functionally most relevant for the processing of faces prior to their selection as targets for upcoming eye movements.

Finally, our data show that visual information processing in high-level visual regions displays the properties necessary to produce the laterality effects that have been reported behaviorally, i.e. different processing of objects and faces when they are presented to the left visual field than when they are presented to the right visual field [20]-[22]. A cortical region is a candidate source of these asymmetries if it shows different functional properties in the two hemispheres, including a preference for contralateral over ipsilateral stimuli. Given that our data show a contralateral bias in both lateral occipital gyrus and fusiform gyrus, any of these regions might underlie the reported behavioral asymmetries.
